# COVID-19 in children and the influence on the employment activity of their female caregivers: A cross sectional gender perspective study

**DOI:** 10.3389/fgwh.2022.1021922

**Published:** 2023-02-01

**Authors:** Catalina Jaime Trujillo, Natalia Herrera Olano, Kevin Rico Gutiérrez, Daniela Medellín, Paola Sánchez, María Lucía Mesa-Rubio, Melisa Sofía Naranjo, Sergio Mauricio Moreno, Carolina Bonilla, Pedro Barrera, Sonia M. Restrepo-Gualteros, Luz Marina Mejia, Olga Lucía Baquero, Juan Gabriel Piñeros, Andrea Ramírez Varela

**Affiliations:** ^1^ Pediatrics Residency Program, Universidad de los Andes and Department of Pediatrics, Hospital Universitario Fundación Santa Fe de Bogotá, Bogotá, Colombia; ^2^ School of Medicine, Universidad de los Andes, Bogotá, Colombia; ^3^ Department of Epidemiology, School of Medicine, Universidad de Los Andes, Bogotá, Colombia; ^4^ Department of Pediatrics Instituto Roosevelt, Bogotá, Colombia; ^5^ Department of Pediatrics Clínica Infantil Colsubsidio, Bogotá, Colombia

**Keywords:** children, adolescent, caregiver, covid-19, employment, gender

## Abstract

**Introduction:**

During the COVID-19 pandemic, women disproportionately assume more unpaid activities, affecting their employment.

**Objective:**

Describe the influence of COVID-19 on the employment of caregivers of children and adolescents from a gender perspective.

**Methods:**

Cross-sectional study in three high-complexity hospitals in Bogotá, Colombia from April 2020 to June 2021. A subsample of the FARA cohort was taken, including those patients with a positive test for SARS-COV2. We took as our analysis category children older than 8 years and younger than 18 years who had a positive SARS-COV2 test, as well as, caregivers of all children with a positive SARS-COV2 test. This subsample was drawn from the FARA cohort. A survey was applied to them. We carried out a descriptive and stratified analysis by age group, educational, and socioeconomic level.

**Results:**

We included 60 surveys of caregivers and 10 surveys of children. The main caregiver in 94.8% of the cases was a female. At the beginning of the pandemic, 63.3% of the caregivers were employed, and 78.9% of those lost their employment. The vast majority of these caregiver were women (96.6%, *n* = 29). A predominance of loss of work activity was documented in caregivers of children in early childhood 66.6% (*n* = 20), with lower education 66.6% (*n* = 20), and from lower strata 56.6% (*n* = 17).

**Conclusion:**

Caregivers of children with COVID-19 with low educational levels and lower socioeconomic conditions, as well as those with children under 5 years showed greater likelihood of employment loss between the interviewed subsample.

## Introduction

In late 2019 a new coronavirus was identified in Wuhan, China (1). In February 2020, the World Health Organization (WHO) termed the disease COVID-19, and the virus causing the disease was named severe acute respiratory syndrome coronavirus 2, also known as SARS-COV2, and on March 12, 2020, the WHO declared COVID-19 a pandemic (1, 2).

Gender inequities refer to avoidable unfair and unnecessary inequalities between men and women (3). During the COVID-19 pandemic, the International Society for Social Pediatrics and Child Health (ISSOP) recognized that actions introduced to reduce the spread of the disease have affected vulnerable populations. By closing schools and day care centers, these populations are exposed to additional stress due to the need for childcare at home and adjustments in daily routines (1).

Traditionally, childcare has been delegated to women, who assume responsibility for their family and home not by choice. This assignment is based on multidimensional factors that give women a specific role in the division of household activities (4). And the International Labor Organization points out that in the labor field there are marked gender inequalities based on regional gender stereotypes, where women are seen as the primary caregivers and men as the breadwinners (5).

Accordingly, during the COVID-19 pandemic, women in Colombia spent twice as much time as men on unpaid childcare and domestic work, a burden that increased with the closing of schools and other measures such as preventive isolation in homes (6). Fewer women than men are employed, and when they are, they are employed informally (6). Data from the National Administrative Department of Statistics (DANE) during 2020 shows that the loss of employment among women has been disproportionately greater than among men (7).

The number of women employed in the second trimester of 2020 was 6.7 million; 2.5 million less than in 2019, and their employment decreased by 27%, which is 9% more than for men (ONU Mujeres 2020). There was also a higher impact on female employment, with a year-on-year contraction of 19.6% in the July to September 2020 trimester and the same trimester of 2019, representing a loss of 1.8 million jobs. This is compared to an 8.1% decline in male employment (8).

We approached the analysis of this problem from the model of social determinants of health (9). These determinants explain how differences in social position contribute to health inequities (9). Structural determinants generate a stratification and division of social classes, and within these determinants are both occupation and gender, which are the starting point for the development of this study (9).

We constructed a conceptual model in which gender is defined by the social conventions, roles, and behaviors established at a cultural level that are responsible for configuring the relationship between men and women, boys, and girls (9).

We based the approach to gender on intersectionality, considering that gender interacts with other dynamics of life, and these have direct implications on a person's comprehensive development (10, 11). Attention should be focused on everything that defines access to rights and opportunities, giving it an approach that makes it possible to identify factors that affect women's lives such as age, ethnicity, socioeconomic strata, educational level, among others (12). Primarily, social models based on gender have a greater burden of negative effects on women (9).

Occupation also determines a position in an individual's hierarchy at the social level according to the person's position in the labor market and its relation to income, the ability to access privileges, creation of social networks, and labor exposures (9). This is one of the determinants that may be affected by gender stereotypes. But there are no studies in Colombia that address this problem; therefore, this study aims to characterize the influence of COVID-19 on children and adolescents based on the labor activity of their caregivers from a gender perspective.

## Materials and methods

We conducted a cross-sectional observational study in three high-complexity hospitals (hospitals with all specialties) in Bogotá between April 2020 and June 2021. The patients included were selected from a cohort called FARA (acute respiratory failure) which was a multicentric cohort study, following up on children and adolescents between 1 month and 18 years of age that were admitted with signs of respiratory distress and therefore at risk of presenting acute respiratory failure. In addition, if they presented acute respiratory failure, follow-up continued at 48 h, at discharge from the institution, and at 30 and 60 days after discharge. It was conducted as part of a supervised collaborative research within the pediatrics residency program at the Universidad de los Andes. To calculate the sample size of the cohort, the overall incidence of acute respiratory failure was calculated in the three institutions where the study was carried out. In addition, the Peduzzi formula, adjusted to a finite population, was used to obtain the minimum sample size necessary to establish associations. A minimum number of 477 individuals was calculated and an additional 20% was included considering the possible loss of patients in a cohort.

In the case of this study, it was not possible to calculate the sample size because there was insufficient information to find statistically significant differences according to sample size, since data about SARS-CoV2 in pediatrics were scarce and there were no studies for a sample calculation in this pathology and age group. A subsample was taken from this cohort, which included children with a positive SARS-COV2 test, regardless of the development of acute respiratory failure.

To collect the information, we designed a survey based on the methodology proposed by the Pan American Health Organization to analyze health conditions from a gender perspective (13). We conducted a comprehension test about the survey and, subsequently, it was applied to all the caregivers and to children over 8 years of age. The survey was administered at least one week after hospital discharge by previously trained researchers. The exclusion criteria were deceased patients.

Approval was obtained from the ethics committee of the School of Medicine of the Universidad de Los Andes, which is recorded in minutes No. 202010271. An amendment was submitted to the institutional review boards to make it possible to study the social impact of COVID-19. We also obtained informed consent from the parents and informed assent from patients older than 8 years of age.

We made a descriptive analysis including the sociodemographic characteristics of children and adolescents and their caregivers, establishing measures of central tendency in quantitative variables and measures of absolute and relative frequency in qualitative variables. We conducted a stratified analysis by age group, educational level of the caregiver, and patient socioeconomic strata concerning the loss of employment activity to make an analysis based on intersectionality. Finally, by evaluating the reasons offered by the respondents in open questions, we determined whether these represented gender stereotypes or not. For this we used the program Stata SE 17.

## Results

**Figure 1:**
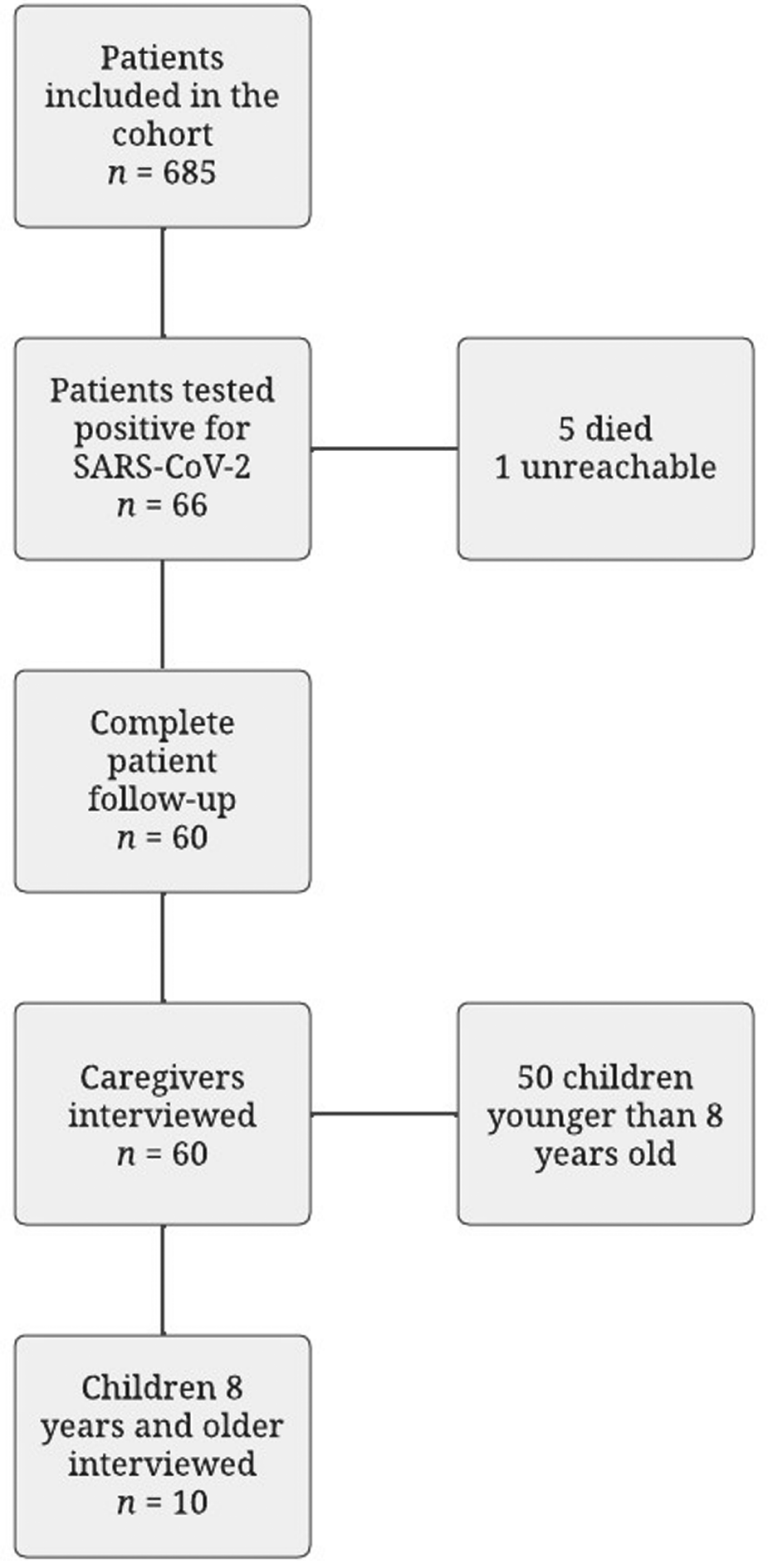
Flowchart for follow-up patients.

A total of 65 patients were identified with a positive test for SARS-COV2. Of these, four patients died, and one was not possible to contact for follow-up because of no answer in the contact numbers after more than 3 attempts. Figure 1 shows the flow chart of patients included in the cohort and the ones included in this subsample.

A total of 10 children (8 years or older) and 60 caregivers were surveyed. The objective was to take into account the children's perspectives in those older than 8 years old, as well as the caregiver's answers.

### Children

**Figure 2:**
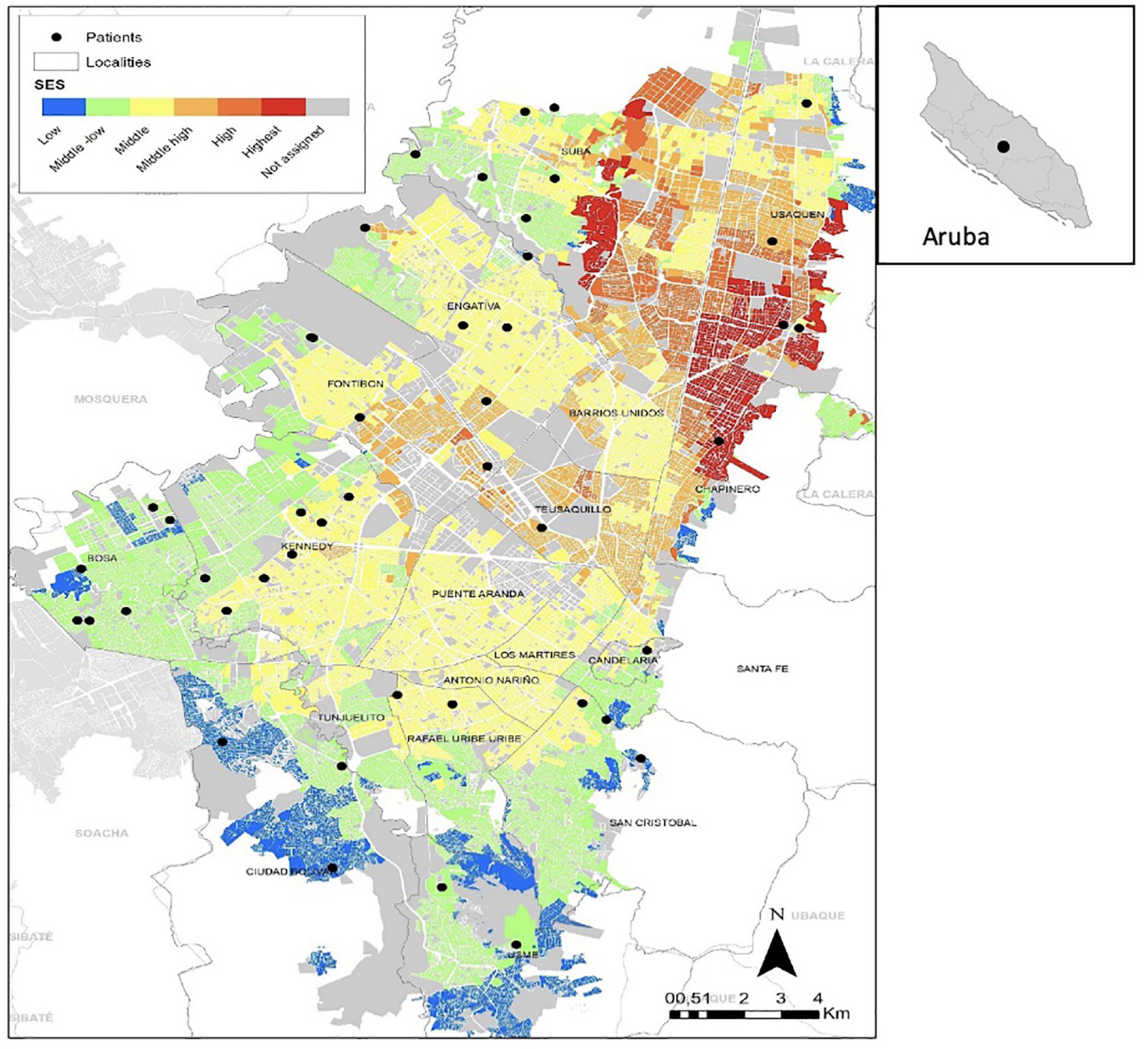
Distribution of housing in bogota by location and socioeconomic Strata and foreing patient.

Table 1 shows the sociodemographic characteristics of the children included. The study sample was made up primarily of children age 0 to 5 years (*n *= 46, 76.7%). The median age of the patients was 1 year. The number of patients and caregivers who lived in Bogotá was 73.3% (*n* = 44). Figure 2 shows their distribution according to locality and socioeconomic strata, which evidences its distribution and how the majority of the patients belonged to the low and middle-low strata (78.4%). One of the patients included lived in Aruba. The rest resided outside Bogotá, including one foreign patient. Although more than half of patients belonged to a very low and low socioeconomic level (*n* = 37, 75.5%, stratum 1–2), few belonged to the subsidized regime of health (*n* = 8, 13.3%).

**Table 1:** Sociodemographic characteristics of children.

Characteristics	*n*	%
**Sex**		
Female	21	35.0
Male	39	65.0
**Age group** ^a^		
Early childhood (0–5 years)	46	76.7
Infancy (6–11 years)	5	8.3
Adolescence (12–18 years)	9	15.0
**Socioeconomic strata** ^b^		
Very low (1)	6	10
Low (2)	31	51.7
Middle low (3)	16	26.7
Middle (4)	4	6.7
Middle high (5)	1	1.7
High (6)	1	1.7
Non-classified (foreigner)	1	1.7
**Health insurance**		
Government subsidized	8	13.3
Contributive	52	86.7
**Family composition**		
Biparental	43	71.7
Monoparental (mother)	14	23.3
Monoparental (father)	3	5

^a^ Age group was established by the Ministry of Health and Social Protection into Early Childhood from 0 to 5 years of age, Infancy from 6 to 11 years of age, and Adolescence from 12 to 18 years of age.

^b^ Socioeconomic strata is defined by the National Department of Statistics (DANE) of Colombia from 1 (very low strata) to 6 (high strata).

### Caregivers

Table 2 identifies the characteristics of the caregivers, where 100% of the caregivers corresponded to a family member; 73.4% (*n* = 44) were between 18 and 39 years of age, with a median age of 31.5 years; and 66.1% (*n* = 39) had a technician or high school education level.

**Table 2:** Sociodemographic characteristics of caregivers.

Characteristics	*n*	%
**Primary caregiver**		
Mother	52	86.6
Grandmother	3	5
Father	3	5
Other	2	3.3
**Age (years)**		
18–29	25	41.7
30–39	19	31.7
40–49	11	18.3
50 or older	5	8.3
**Educational level**		
Middle school	7	11.9
High school	15	25.4
Technician	24	40.7
Higher education	13	22.0
Education level not provided	1	1.6

**Figure 3:**
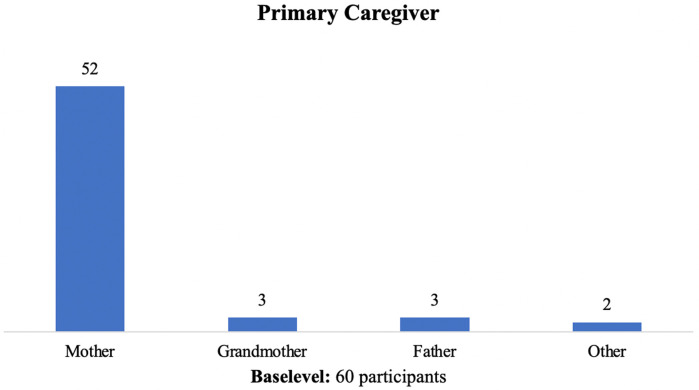
Who is the primary caregiver of children with COVID-19?.

As shown in Figure 3, in 92% of the cases, the main caregiver is female: the mother in 86% (52) of the cases and the grandmother in 5% (3) and the caregiver is the father in 5% (3) of the cases, even though most of the families are biparental families (71.7%).

### Caregivers' employment

**Figure 4:**
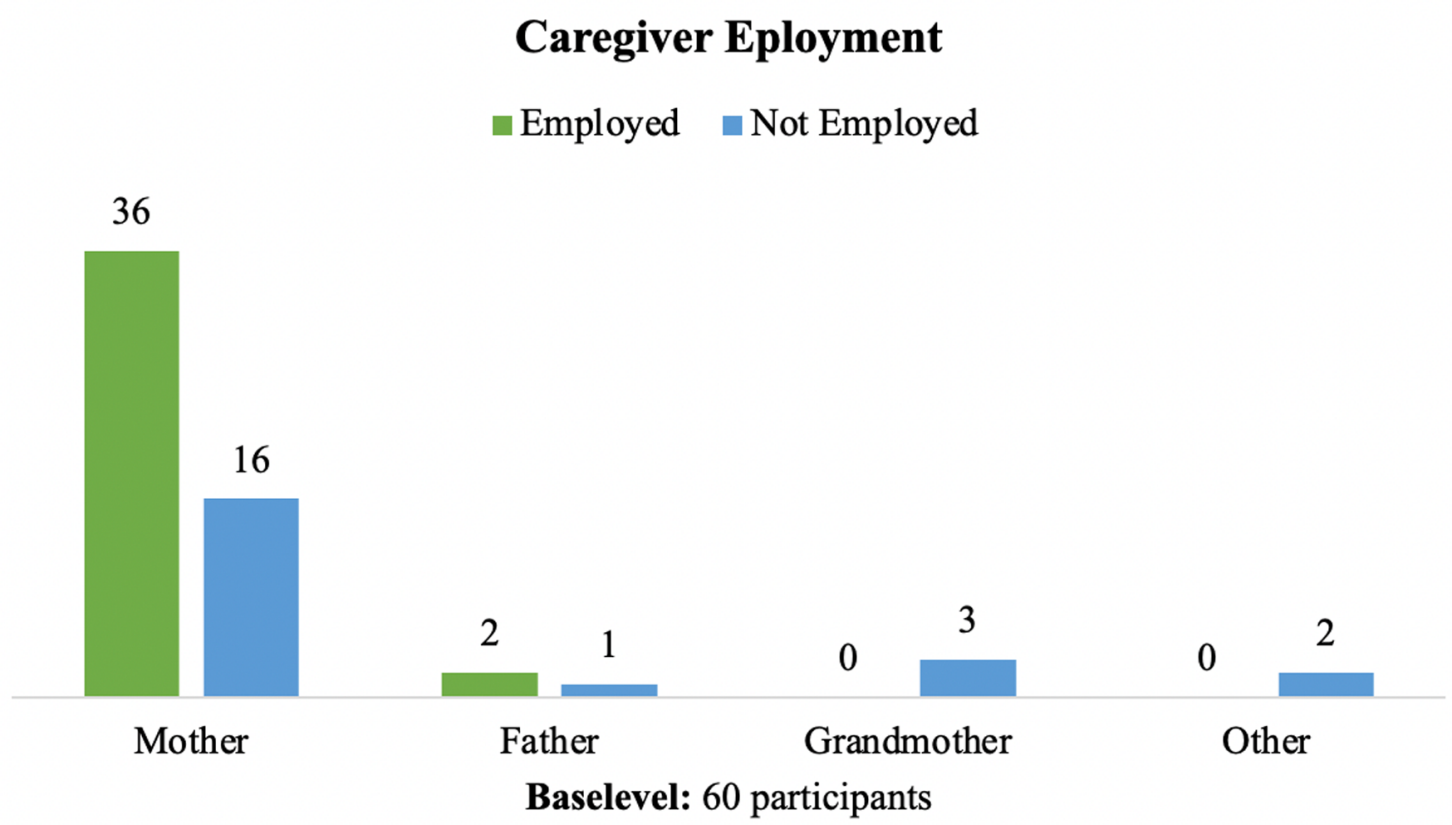
Caregiver work activity at the onset of COVID-19 pandemic.

**Figure 5:**
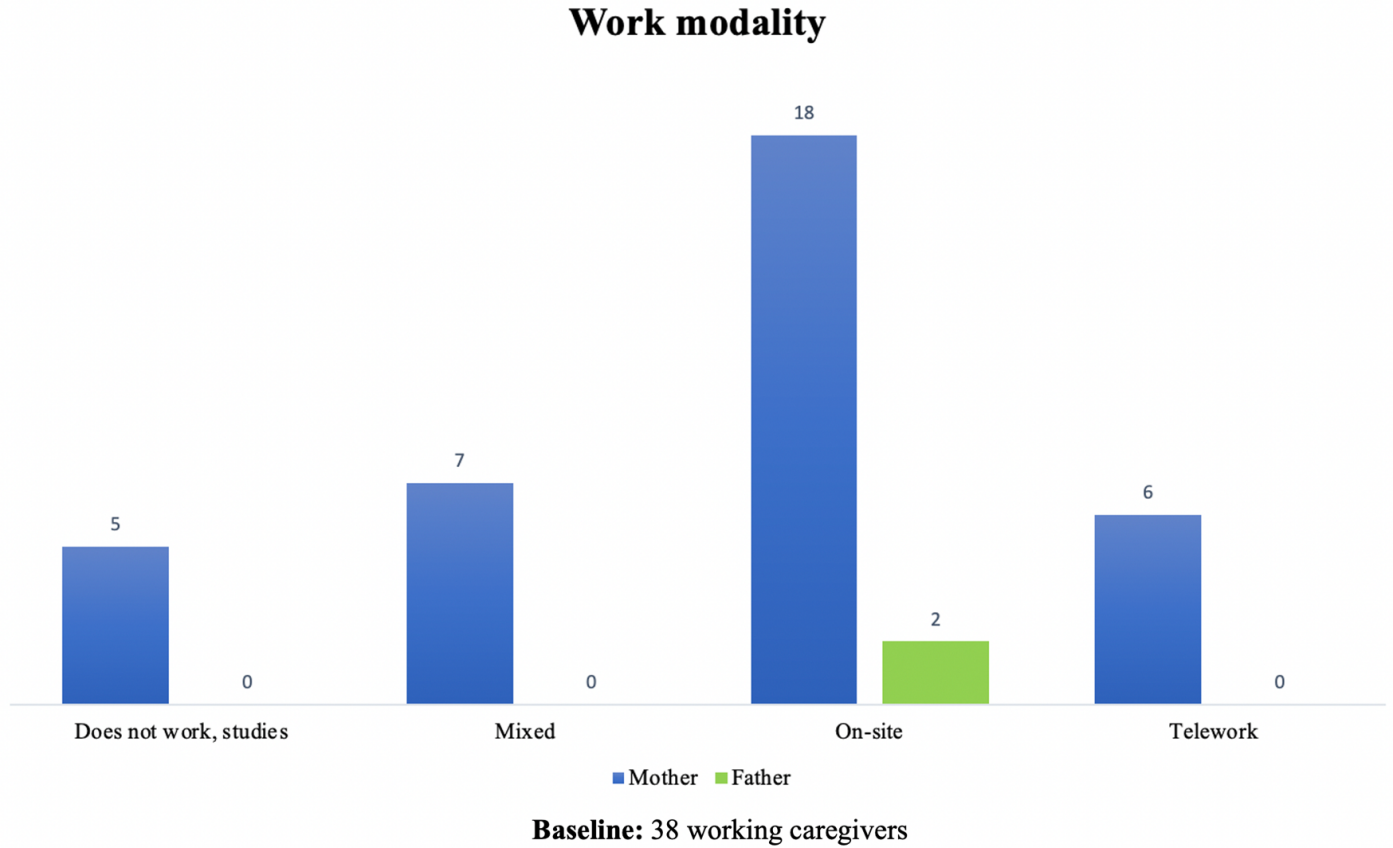
Work modality by caregivers.

Of the primary caregivers, 63.3% (*n* = 38) stated that they were working or studying at the beginning of the pandemic. Figure 4 shows the distribution of work according to the type of caregiver for each child, where 36 of the caregivers were women and 2 were men. Regarding the work modality, 52.63% (*n* = 20) had to work on-site, compared to 15.9% (*n* = 6) with the opportunity to telework. In 18.42% (*n* = 7) of the cases, they worked in a mixed modality, and in 13.16% (*n* = 5) they only studied. According to Figure 5, half of the mothers had to work on-site, this was related to a higher risk of work stability.

Of those caregivers who were working at the beginning of the pandemic, 63.3% (*n* = 19) had to stop working or studying because of the pandemic while 36.6% (*n* = 11) had to stop working because of the child's health condition, for a total of 78.9% (*n* = 30) of work loss. Among those who lost their work activity, the vast majority were women (96.6%, *n* = 29).

In the stratified analysis, a predominance of loss of work activity was documented in those caregivers who were employed on-site (53.3%, *n* = 16), caregivers of children in early childhood 66.6% (*n* = 20), those with a technical or high school education 66.6% (*n* = 20), and those from lower strata 56.6% (*n* = 17).

In addition, 41.67% (*n* = 25) of caregivers expressed a need for economic support, 36.7% (*n* = 22) needed spiritual and social support, and 25% (*n* = 15) needed psychological support, with a predominance of need for economic support in those who experienced loss of employment due to the pandemic.

**Figure 6:**
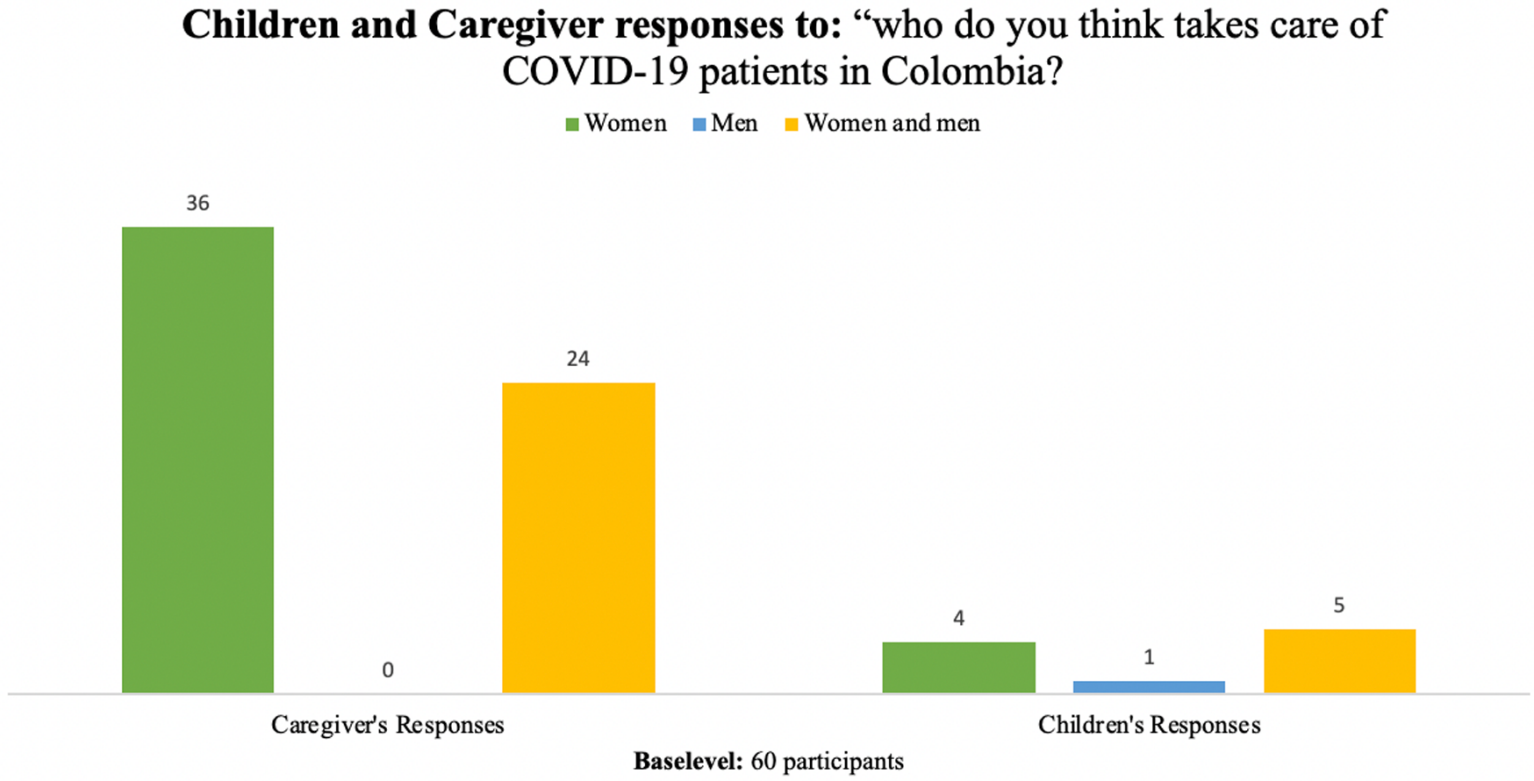
Children and caregiver responses to question “who do you Think Takes Care of COVID-19 Patients Colombia?”.

Figure 6 shows who the children respondents and caregiver respondents believe takes care of COVID-19 patients in Colombia. This shows the difference in perceptions between children and caregivers, where caregivers consider taking care of COVID-19 patients is more attributed to women, while children gave more importance to a shared responsibility between men and women.

### Children beliefs

Of children and adolescent respondents, we found that five of them (50%) believed that COVID-19 patients in Colombia were cared for by women and men, four (40%) mentioned that care was done by women only, and one of them (10%) mentioned care was done by men.

These answers represent a gender stereotype. For example, regarding women as caregivers, the children and adolescents mentioned that “women are the ones who care the most, they are more careful and more attentive. In addition, there were no men among the doctors and nurses”. “The mothers know all the remedies and women are more cautious” and “women are more service-oriented.” Regarding men as caregivers, the children and adolescents said, “They are more responsible.”

On the other hand, those children and adolescents who responded that caregiving is done by both women and men do not appear to have an ingrained gender stereotype, giving answers such as: “Both help, there is no difference between the two genders”, “Because men and women take care of everyone equally,” and “There is no difference between a woman and a man.”

### Caregivers' believings

Of the caregiver respondents, we found that 60% (36) believed that the care of patients with COVID-19 in Colombia is carried out by women, 40% (24) responded that care was done by men and women, while none responded that it was only men.

Like the children and adolescent respondents, all adults who responded that women are mostly the ones who care for patients with COVID-19 gave answers that reflect gender stereotypes. For example, “We women worry more and take more care of the sick” and “They are the ones who should be aware, they know more about caring for children and the sick.” Those who responded that care is carried out by men and women did not reflect gender stereotypes, stating: “Both have the same rights, both take care equally” and “We are all committed to the care of family members and the community.”

## Discussion

This study is one of the few that has evaluated the pandemic's influence on the employment activity of caregivers of children with COVID-19. We found that the activity of female caregivers of children and adolescents was affected during the pandemic by abandoning or losing their jobs to assume a caregiving role in the home, either because of pandemic-related factors or because of a SARS-COV2 infection of their children.

We found that in almost 92% of the cases the main caregiver was a woman, represented by mothers and grandmothers. This finding is similar to data reported at a national level, in which 77% of domestic and unpaid care work is performed by women (14) and even surpasses data previously recorded in Colombia in which 84% of caregivers were women (5). This could be explained by culturally ingrained gender stereotypes that imply the presence of hegemonic models of femininity and masculinity, in which women assume the additional responsibilities corresponding to domestic and unpaid care activities.

Regarding the employment activity of caregivers, 63% of caregivers were working at the onset of the pandemic. Of those caregivers, almost 80% lost their jobs; 63.3% because of the pandemic and the rest because of the health status of their children. And 96.6% of those were women. This evidence is consistent with the existing literature regarding a setback of more than 30 years, decreasing the rate of labor participation of women by 16 percentual points during the pandemic, and increasing the gender gaps in unpaid work (14–16). Particularly, according to the DANE, Colombia ranks 22nd out of 153 countries in the Global Gender Gap Index, which measures the existing gender gap in labor and economic participation (14, 16).

These results can be explained as a reflection of the existing gender inequalities at the level of labor activity, with women under greater informality of labor conditions, which makes them more vulnerable to unemployment in the context of a health emergency.

In addition, the study documented that labor loss predominated in families with children in early childhood, belonging to low socioeconomic and cultural strata in which survival requires on-site work. Evidence supports these findings, as more care is required in early childhood due to the child's greater dependence and vulnerability during this stage (17).

With the findings of this study, it is possible to demonstrate that COVID-19 in children and adolescents can have a major impact on the work activity of their caregivers. The presence of illness implies the need for adjustment, both during the illness and during recovery, and specifically in the context of a pandemic, in which governments were forced to dictate measures such as quarantine.

Recognizing the family environment and the caregivers' conditions will help avoid value judgments toward families and those who care for sick children. This will also help health care providers recognize the importance of knowing the circumstances of each family, thus improving the quality and comprehensiveness of care.

The strengths of this study are its development under the creation of a conceptual model with a gender perspective related to the effect of the disease on the primary caregiver. A clinical sample of COVID-19 cases was obtained in Bogotá with a clinical presentation of respiratory symptoms associated with respiratory distress and, in some cases, acute respiratory failure, which is relevant because there is a large proportion of asymptomatic infections in pediatric cases (18) and the most severe spectrum of the disease was included, which implies a greater burden of care. This sample includes a population from three high-complexity hospitals including different socioeconomic levels, which allows us to analyze the social implications of the disease.

Limiting this study is the impossibility of assessing the presence of comorbidities in these patients since chronic disease is an additional factor that could impact care needs. To overcome this limitation, this variable could have been included in the collection instrument to analyze the information in these patients. It was also not possible to follow up with the caregivers over time. This counteracts the evaluation over time of what happened with these caregivers and their jobs. This opens opportunities for research to evaluate how these families evolve during the pandemic.

According to the results, we conclude that there is an evident gender inequity in the care of children with COVID-19 as well as in the influence on the work activity of these caregivers. Therefore, it is necessary to design processes to sensitize health professionals to the importance of identifying social determinants and for families to know how this context determines the possibilities of care in a hospital environment. We also conclude that the measures put in place due to a health emergency should incorporate the gender perspective and an intersectional approach in order to lessen the negative effect on vulnerable populations.

## Data Availability

The raw data supporting the conclusions of this article will be made available by the authors, without undue reservation.
